# Diatom Biogeography, Temporal Dynamics, and Links to Bacterioplankton across Seven Oceanographic Time-Series Sites Spanning the Australian Continent

**DOI:** 10.3390/microorganisms10020338

**Published:** 2022-02-01

**Authors:** Nine Le Reun, Anna Bramucci, James O’Brien, Martin Ostrowski, Mark V. Brown, Jodie Van de Kamp, Levente Bodrossy, Jean-Baptiste Raina, Penelope Ajani, Justin Seymour

**Affiliations:** 1 Climate Change Cluster, University of Technology Sydney, Ultimo, NSW 2007, Australia; nine.m.lereun@student.uts.edu.au (N.L.R.); anna.bramucci@uts.edu.au (A.B.); james.obrien@student.uts.edu.au (J.O.); martin.ostrowski@uts.edu.au (M.O.); jean-baptiste.raina@uts.edu.au (J.-B.R.); 2 School of Environmental and Life Sciences, The University of Newcastle, Callaghan, NSW 2308, Australia; oceanmicrobes@gmail.com; 3 Oceans and Atmosphere, Commonwealth Scientific and Industrial Research Organisation, Battery Point, TAS 7004, Australia; jodie.vandekamp@csiro.au (J.V.d.K.); lev.bodrossy@csiro.au (L.B.); 4 School of Life Sciences, University of Technology Sydney, Ultimo, NSW 2007, Australia; penelope.ajani@uts.edu.au

**Keywords:** diatoms biogeography, seasonality, time-series, Australia, marine bacteria, co-occurrence

## Abstract

Diatom communities significantly influence ocean primary productivity and carbon cycling, but their spatial and temporal dynamics are highly heterogeneous and are governed by a complex diverse suite of abiotic and biotic factors. We examined the seasonal and biogeographical dynamics of diatom communities in Australian coastal waters using amplicon sequencing data (18S-16S rRNA gene) derived from a network of oceanographic time-series spanning the Australian continent. We demonstrate that diatom community composition in this region displays significant biogeography, with each site harbouring distinct community structures. Temperature and nutrients were identified as the key environmental contributors to differences in diatom communities at all sites, collectively explaining 21% of the variability observed in diatoms assemblages. However, specific groups of bacteria previously implicated in mutualistic ecological interactions with diatoms (Rhodobacteraceae, Flavobacteriaceae and Alteromonadaceae) also explained a further 4% of the spatial dynamics observed in diatom community structure. We also demonstrate that the two most temperate sites (Port Hacking and Maria Island) exhibited strong seasonality in diatom community and that at these sites, winter diatom communities co-occurred with higher proportion of Alteromonadaceae. In addition, we identified significant co-occurrence between specific diatom and bacterial amplicon sequence variants (ASVs), with members of the *Roseobacter* and *Flavobacteria* clades strongly correlated with some of the most abundant diatom genera (*Skeletonema*, *Thalassiosira*, and *Cylindrotheca*). We propose that some of these co-occurrences might be indicative of ecologically important interactions between diatoms and bacteria. Our analyses reveal that in addition to physico-chemical conditions (i.e., temperature, nutrients), the relative abundance of specific groups of bacteria appear to play an important role in shaping the spatial and temporal dynamics of marine diatom communities.

## 1. Introduction

Diatoms (Bacillariophyta) are one of the most abundant and diverse groups of marine phytoplankton, and are responsible for approximately 20% of global marine primary productivity [1,2]. These unicellular photosynthetic eukaryotes constitute the base of the marine food web [3,4], and are especially abundant in coastal regions and polar nutrient-rich waters [5,6,7]. They also play an important role in the biological pump, as their silicified frustules rapidly sink to the ocean’s seafloor and contribute to carbon sequestration [8]. Given their ecological and biogeochemical importance, identifying the factors that influence diatom assemblage structure and diversity over space and time is essential for understanding the processes governing marine ecosystem productivity and function.

Diverse environmental factors have been demonstrated to influence diatom composition and abundance [9,10,11]. For example, temperature and light exposure have repeatedly been shown to be determinants of diatom abundance and diversity over space and time and contribute to their seasonality and bloom dynamics [12]. Diatom community composition is also determined by nutrient availability (e.g., nitrogen, phosphorus and iron) and many diatom species can rapidly grow in response to increase in nutrient concentrations (e.g., during upwelling events). This can lead to bloom events that last for days to weeks and sometimes induce a succession of small faster-growing species (e.g., *Thalassiosira*, *Skeletonema*, *Pseudo-Nitzschia*) initially dominating the consortia, followed by larger diatoms (e.g., *Rhizosolenia*, *Leptocylindrus*) [12,13].

Ecological processes can also influence diatom communities through interactions with other organisms [14,15,16], whereby virus infection and grazing by zooplankton can have a negative impact on diatom abundance [15]. Alternatively, recent work has also revealed the potential beneficial impacts of ecological interactions between diatoms and heterotrophic bacteria, which are often underpinned by reciprocal chemical exchanges, including the bacterial provision of vitamins (e.g., B12) and other micro-nutrients (e.g., iron) [17,18]. For example, *Asterionellopsis glacialis* can adapt its metabolic response to secrete specific metabolites (rosmarinic acid and azelaic acid), which promote the attachment of beneficial bacteria and inhibit the growth of opportunistic ones [19]. Similarly, the roseobacter *Sulfitobacter* produces the algal growth hormone indole-3-acetic-acid (IAA), which promotes *Pseudo-nitzschia* growth [20]. While in the beneficial association between *Phaeodactylum tricornutum* and the Alphaproteobacterium *Stappia*, the bacterium uses fatty acid synthesized by *P. tricornutum* and in turn enhances the diatoms growth by upregulating growth-related genes (i.e., DNA replication, cell division, transcription) [21].

Determining how both biotic and abiotic factors shape the spatial and temporal dynamics of marine diatom communities is important for understanding and even predicting shifts in diatom community structure, abundance and changes in geographical boundaries driven by climate change. The biogeographic distribution patterns of diatoms have been characterised through large-scale sampling efforts (e.g., Tara Oceans, Continuous Plankton Recorder) [22,23], revealing that the abundance and composition of diatom communities are highly variable in space [24]. For example, the Tara Ocean expedition demonstrated that many diatom genera, such as *Chaetoceros*, *Fragilariopsis* or *Thalassiosira* are globally distributed, but their diversity and abundance are highly variable depending on latitude [6]. Diatom diversity within the photic zone tends to decrease toward the poles (equator), due to temperature gradients and associated nutrient availability [25]. Oceanographic circulation patterns also greatly influence both the dispersal of diatom communities and can promote bloom events through mixing of deep water [6,26]. Additionally, while many previous studies have demonstrated that physicochemical conditions influence diatom communities, very little is known about the potential role of bacterial communities in shaping diatom biogeography, which is a notable gap given evidence from laboratory studies that bacteria can influence the growth of many diatom species [21,27,28]. Although spatial studies of diatom communities have provided important insights into their diversity and distribution, these approaches are typically a ‘snap-shot’ in time and do not consider temporal dynamics at each location. Seasonal shifts in diatom diversity and composition may therefore remain undetected or underestimated by many of these global sampling efforts [29].

Temporal studies at specific locations (mostly coastal) [30,31,32] have demonstrated that diatom communities exhibit strong seasonal patterns, with higher biomass generally observed during winter and spring, typically as consequence of seasonal variability in nutrients and temperature [32,33]. Shorter temporal studies with higher resolution (daily timescales) have also identified very rapid changes in diatom community assemblages [29]. Alternatively, while there is evidence that diatoms shape bacterial communities composition mainly through the release of different organic compounds during bloom events [34,35] the potential reciprocal influence of bacteria on diatom communities in the ocean remains largely unknown. Furthermore, most time-series studies are geographically restricted to specific, long-term sampling sites [36,37,38], meaning that they are only representative of specific latitude and physico-chemical conditions. Thus, simultaneous characterisation of the spatial and temporal dynamics of diatom communities is required to more clearly define the ecological processes that are the most important for determining diatom community structure and abundance.

Here, we performed a spatial and temporal characterisation of the diversity of diatom communities across a continent-scale network of seven oceanographic time-series sites, spanning over 30 degrees of latitude (ranging from tropical to temperate environments) and a period of 5 years. Using this dataset, we characterise the key abiotic and biotic determinants of diatom community composition, including the potential influence of marine bacterial communities.

## 2. Methods

### 2.1. Sampling Sites, Collection and Processing

**Figure 1:**
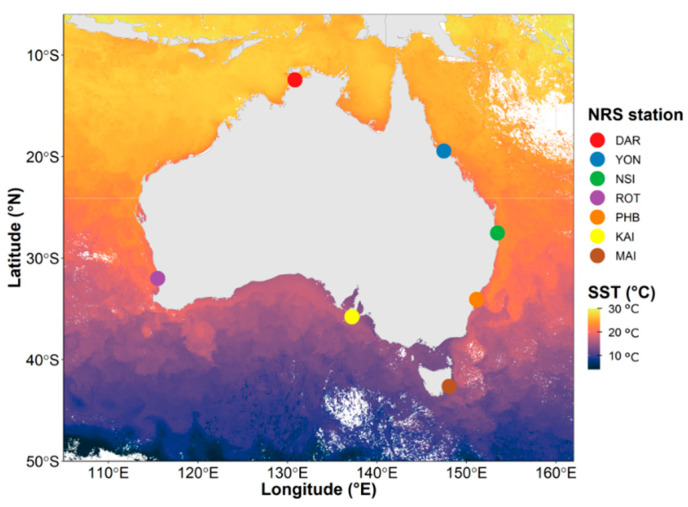
Variability of Sea Surface Temperatures Across the Seven National Reference Stations (NRS) Around Australia. The seven stations span 30-degree latitude and 20-degree longitude: from the northernmost site clockwise around Australia: Darwin (DAR), Yongala (YON), North Stradbroke Island (NSI), Port Hacking (PHB), Maria Island (MAI), Kangaroo Island (KAI) and Rottnest Island (ROT). Ocean colours represent the Sea surface temperature (°C) of a single date from MODIS ocean colour (https://oceancolor.gsfc.nasa.gov/l3/ (accessed on 23 August 2021)).

Water samples were collected (monthly or quarterly), between 2015 and 2019, from a network of 7 oceanographic time-series sites comprising the Integrated Marine Observing System (IMOS) National Reference Station (NRS) (https://imos.org.au/facilities/nationalmooringnetwork/nrs (accessed on 5 April 2020) network. These sites span Australian coastal shelf environments from temperate to tropical latitudes within the Pacific, Southern and Indian Oceans (Figure 1, Table S1). Each of these sites is characterised by discrete oceanographic characteristics and physicochemical conditions [39,40]. For instance, the northernmost site Darwin (DAR) is located within the Arafura Sea and is characterised by monsoonal climate along with the highest sea surface temperatures of all sites (25 to 31 °C, Table S2). In contrast, the southernmost site at Maria Island (MAI) is characterised by a temperate climate and substantially cooler water temperatures (11 to 20 °C, Table S2). Yongala, located in the Coral Sea, is also characterised by tropical and monsoonal climate with a slightly lower average temperature (~28 °C) than Darwin. The Port Hacking (PHB) and North Stradbroke Island (NSI) sites are both located in the Tasman Sea, are characterised by subtropical climatic conditions (~21 °C and 24 °C, respectively), and are impacted by the Eastern Australian Current (EAC), which is a rapidly moving western boundary current transporting warm waters from the Coral Sea into the Tasman Sea [41] (Figure 1). Rottnest Island (ROT) is located off the southwestern coast of Australia, within the Indian Ocean and is influenced by the southward flow of warm water from another boundary current, the Leeuwin Current [42] (Figure 1). Water temperatures at this sub-tropical site range between 17 and 22 °C. Finally, Kangaroo Island (KAI) is located off the southern coast of Australia in the Southern Ocean and is characterised by cool temperate conditions, with seawater temperatures ranging between 12 and 19 °C.

At each site, surface temperature (°C) and depth (m) were recorded using a Conductivity-Temperature-Depth (CTD) Seabird SBE19plus profiler. Water samples were collected using Niskin bottles, from a range of depths depending on site characteristics (Table S1), ranging from surface waters (2 m) to depths of up to 100 m. Nutrient concentrations, including silicate (µmol L^−1^), phosphate (µmol L^−1^), nitrate + nitrite (µmol L^−1^) and ammonium (µmol L^−1^), were obtained from 30 mL unfiltered seawater and measured using a Seal AA3HR segmented flow auto-analyser and a JASCO FP2020 fluorescence detector for ammonia (Seal AutoAnalyzer Application method no G-327-04 Rev 4) [43]. For microbial sample collection, 2 L of seawater was filtered through a 0.2 µm pore Sterivex GP filter using a peristaltic pump, which was subsequently stored at -80 °C. Daylength (h) was calculated using the *daylength* function from the ‘geosphere’ R package with calculation based upon latitude and day of the year. In total, over a five-year period (2015–2019), 212 samples from surface water were analysed for characterisation of diatom and bacterial communities in this study (Tables S2 and S3).

### 2.2. DNA Sequence Processing

DNA extraction and sequencing were performed according to the standardised protocols of the Australian Microbiome Initiative (https://www.australianmicrobiome.com/ (accessed on 5 April 2020)) and as previously detailed in Brown et al., (2018) [39]. Briefly, DNA was extracted and purified using the PowerWater Sterivex Isolation Kit and stored at −80 °C. Amplicon sequencing targeting the eukaryotic 18S rRNA V4 gene (TAReuk454FWD1: CCAGCASCYGCGGTAATTCC; TAReuk-Rev3: ACTTTCGTTCTTGATYRATGATCTRYATC) and bacterial 16S rRNA V1-V3 gene (27f: AGAGTTTGATCMTGGCTCAG; 519r: GWATTACCGCGGCKGCTG) was used to characterise patterns in diatom diversity and potential ecological associations with bacteria, respectively. All sequencing was performed at the Ramaciotti Centre for Genomics (UNSW Sydney, Australia), using dual indexed paired end sequencing on the Illumina MiSeq platform [42]. All unprocessed eukaryotic and bacterial paired end Illumina R1 and R2 reads were downloaded directly from the Bio Platforms Australia Marine Microbes data portal on August 2020 (https://data.bioplatforms.com/organization/australian-microbiome (accessed on 4 August 2020)).

Each sequencing plate was run through the DADA2 pipeline for both diatom (18S rRNA gene) and bacteria (16S rRNA gene) (https://github.com/martinostrowski/marinemicrobes/tree/master/dada2 (accessed on 5 May 2021)). Briefly, the *R*-package DADA2 (version 1.14.0) was used to remove primers using cutadapt [44], trim low quality terminal ends (eukaryotic 18S rRNA trunc lengths R1= 200; R2= 195; bacterial 16S rRNA trunc lengths R1= 255; R2= 250), denoised, merged, and removed chimeras from the dataset (minFoldParentOverAbundance=4) to avoid flagging intra-genomic variants [44]. All ASV tables for the project were then combined. The eukaryotic ASV table was quality filtered to remove all reads <0.0001% within any given sample and the bacterial table was quality filtered to remove all ASVs present <0.00001% of any given sample, then each dataset underwent the collapse no mismatch step. Eukaryotic 18S rRNA ASVs were assigned using Protist Ribosomal References database (PR2) and a bootstrap cut-off of >50%. Taxonomic classification of bacterial 16S rRNA ASVs was performed using a naïve Bayes classifier based on SILVA 138.1 and a bootstrap cut-off >50% (10.5281/zenodo.4587955) [45,46]. To normalize samples and account for variable total read depth between samples, ASV abundances were then rarefied to 20,000 reads for both 18S and 16S dataset using the *rrarefy* function from the ‘vegan’ R package.

### 2.3. Statistical Analysis

All statistical analyses were performed using R (4.0.5, Vienna, Austria) programming software and SPSS (v25.0., Armonk, NY: IBM Corp). Samples collected between 2012 and 2014 were omitted from these analyses due to several abnormally long (4–5 month) gaps between successful sampling periods during these years (cf. Figure S1). Additionally, all statistical analyses were performed on surface water samples as it was the only common depth sampled across all sites (cf. Tables S3 and S4 for list of samples and metadata used). Alpha diversity and richness (Shannon and Chao1 indexes) within diatom communities were calculated on rarefied ASVs data (to 20,000 reads) using the ‘phyloseq’ R package [47], with significance tested using the Wilcoxon rank sum test from ‘stats’ R package. Community level differences between sites were determined by permutational multivariate analysis of variance (PERMANOVA) of Bray-Curtis dissimilarities using the *adonis* function from the R package ‘vegan’ and adjusted *p*-values using a Bonferroni-Hochberg correction [48]. For the purpose of our analyses, we defined a 4-fold increase in relative abundance of ASVs between successive samples as a significant “peak” in abundance.

To identify and visualise the biotic and abiotic factors potentially influencing the diatom community structure, we used Spearman’s correlation and Canonical Correspondence Analysis (CCA). The Mantel statistic (Spearman’s rank correlation) was used to test if bacterial community and which environmental variables (silica, temperature, phosphate, nitrate, nitrite, ammonium, day length, salinity) had a significant relationship with diatom ASVs for each sample and was performed using the *mantel* function from the ‘vegan’ R package. Dissimilarity between samples was calculated based on the Euclidean distance for environmental data and Bray-Curtis distance for diatom community data using the *vedgist* function. Prior to this test, environmental variables were square root transformed and any samples missing required environmental data were removed from the analysis in order to have fully overlapping matrices between the environmental data and the diatom ASVs. Kruskal-Wallis and pairwise comparison Wilcoxon rank sum test analyses were also used to test if there was any statistical difference in environmental variables (silica, temperature, phosphate, nitrate ammonium, salinity, daylength) between sites.

To assess significantly differentially abundant diatom ASVs between sites, we used an analysis of compositions of microbiomes with bias correction (ANCOM-BC) approach with the *ancombc* function from the R package ‘ANCOM-BC’ (v1.0.5). ANCOM-BC uses raw read counts and is based on a log-linear model which accounts for sampling fraction across samples by introducing a sample specific offset term into a linear-regression [49]. The adjusted *p*-value method was set to “BH” (Benjamin-Hochberg) and all other parameters were left as the default (zero_cut = 0.9, meaning that ASVs that are not present (=0) 90% of the time are removed from the analysis). Abundance of all ASVs were compared within one site against all the others (e.g., DAR vs. all other sites, PHB vs. all other sites etc.). This analysis determined which ASVs are significantly differentially abundant at each site compared to all other sites.

To investigate the extent of seasonality in the temporal dynamics of diatom communities, we compared the intra- (yearly) and inter-annual (season) similarity among diatom assemblages at North Stradbroke Island, Porth Hacking and Maria Island, which were the three sites with the most complete datasets between 2017 and 2019 (cf. Figure S1). We calculated Bray-Curtis (BC) similarities among diatom communities at all sites by grouping sample by month and year: Autumn-Winter (March to August) and Summer-Spring (September to February) to obtain 6-month and yearly BC similarities. We then compared similarities between adjacent 6-month interval (Autumn-Winter vs Summer-Spring) and yearly interval (Autumn-Winter vs Autumn-Winter) using Kruskal-Wallis test (*p* < 0.05) in SPSS software.

Analysis of co-occurrence patterns among diatoms (18S rRNA gene amplicons) and bacteria (16S rRNA gene amplicons) were determined using MINE clustering analysis, as described in Needham and Fuhrman (2016) [50]. Briefly, only surface seawater samples common to both diatom and bacteria ASVs were considered and, for both diatom and bacteria, only ASVs with a relative abundance higher than >0.5% in at least one sample were included in the analysis (*n* = 202). Environmental variables (temperature, day length, phosphate, silicate, nitrate and ammonium) were square root transformed. Here, MINE results were filtered by total information coefficient (TIC) values greater than 0 and a maximal information coefficient (MIC) value > 0.24 (corresponding to a *p* < 0.0001 for *n* = 220) to indicate the presence of significant relationships. Then, to retain only the significant interactions, MINE results (TIC*_e_* >0, MIC*_e_* ≥0.024) were pulled from the initial file and run through a MINE allPairs comparison. Network visualisation was limited to only diatom ASVs that had a significant positive (LR >0.7) co-occurrence with any bacterial ASV and was performed using the Organic Layout of the Cytoscape software [51]. 

## 3. Results

### 3.1. Regionally Discrete Environmental Patterns and Diatom Communities

**Figure 2:**
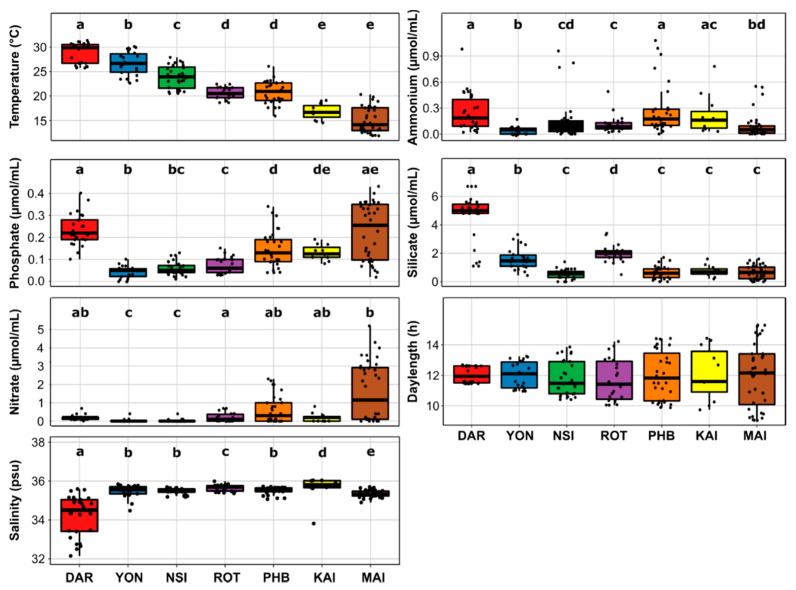
Variability of Environmental Variables Across the Seven Time-Series Sites. Lower and upper limits on each boxplot represent the first and third quartile (25th and 75th percentiles), the median is represented by the solid black line within the box. The whiskers represent the largest and smallest value no further than 1.5 times Interquartile Range (IQR). Different letters (a, b, c, d, e) indicate a significant difference between the associated means (Wilcoxon rank sum test *p* < 0.05). Darwin had 26 datapoints (only 25 for phosphate and nitrate analysis), Yongala 22, North Stradbroke Island 26 (only 25 for ammonium), Port Hacking 31 (30 for ammonium), Maria Island 36, Kangaroo Island 11 and Rottnest Island 22. Each site is represented by a different colour.

All physicochemical parameters considered (temperature, salinity, phosphate, ammonium, nitrate and silica), except day length, were significantly different between two or more of the seven NRS sites (Figure 2). Water temperature was the environmental parameter that most significantly differed between all sites, with the exception of Rottnest Island and Port Hacking, which both had average surface seawater temperatures of 21 °C (Kruskal-Wallis, *p* < 2.2 × 10^−16^; Figure 2, Tables S2 and S5). Across all years sampled, the highest water temperatures were observed at the northernmost site in Darwin (28.7 ± 2.2 °C) and the lowest temperatures were observed at Maria Island (15.1 ± 2.6 °C), which was significantly colder than all other sites (Kruskal-Wallis, *p* < 2.2 × 10^−16^; Figure 2, Tables S2 and S5). Maria Island also had significantly higher phosphate and nitrate levels (Kruskal-Wallis, *p* < 2.2 × 10^−16^ and *p* = 3.102 × 10 ^−11^) compared to other sites, apart from Darwin and Kangaroo Island (Figure 2, Tables S2 and S5). Darwin was characterized by significantly higher concentrations of silicate compared to all other sites (Kruskal-Wallis, *p* < 2.2 × 10^−16^; Figure 2, Tables S2 and S5).

Within surface waters, a total of 1061 diatom ASVs were identified across 212 samples. Both diatom diversity and richness significantly varied between sites (Kruskal-Wallis, *p* < 0.001; Figure S2 and Table S6). Indeed, diatom community diversity at lower latitudes (Darwin and Yongala) was on average 1.3-times higher (Shannon’s index ~3.3, *p* < 0.05; Figure S2, Table S7) than diversity at mid-high latitudes sites corresponding to more temperate environments (Port Hacking, Maria Island and Kangaroo Island) (Shannon’s index ~2.5, *p* < 0.05; Figure S2, Table S7). Following a similar pattern, diatom community richness (Chao1 index ~120, *p* < 0.05; Figure 2, Table S7) was 1.8-times higher at the lower latitude stations (Darwin and North Stradbroke Island) relative to higher latitude locations (Maria Island and Kangaroo Island) (Chao1 index ~67; Figure 2, Table S7).

**Figure 3:**
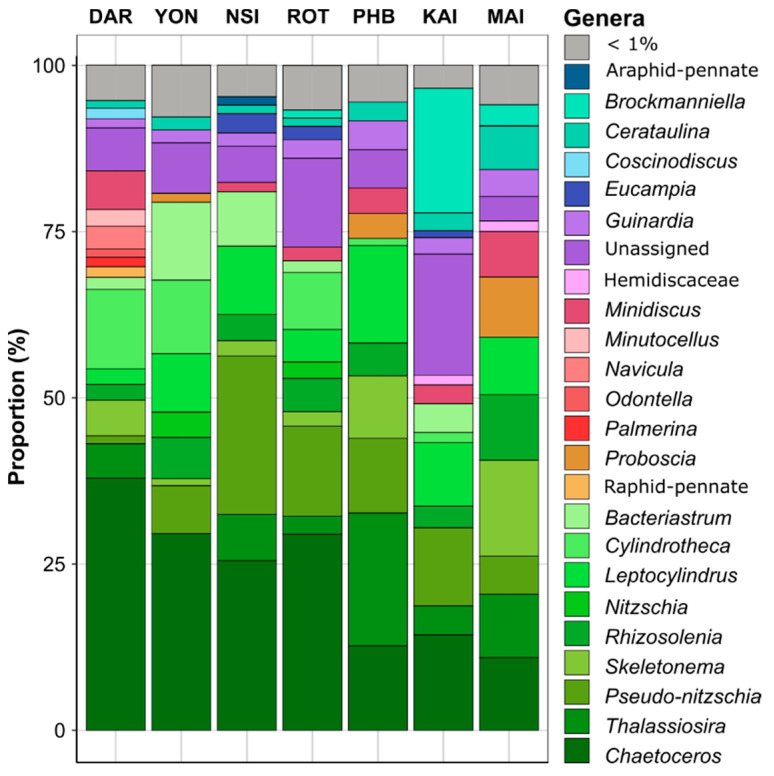
Marine Diatom Composition of Surface Waters (2 m) Collected from Seven National Reference Stations around Australia. Relative abundance (%) of the most abundant diatom genera (>1% threshold) across all sampling points between 2015–2019, using 18S rRNA gene as a phylogenetic marker (DAR: 28 samples, YON: 23 samples, NSI: 40 samples, ROT: 25 samples, PHB: 38 samples, KAI: 12 samples, MAI: 46 samples). Non-italic text corresponds to higher taxonomy levels. The full dataset is presented in Figure S1.

Diatom community structure differed significantly between sites (PERMANOVA and all pair-wise comparison, *p* < 0.001; Table S8). Across all samples, ASVs belonging to the *Chaetoceros* genus were the most abundant diatoms observed. This genus was particularly abundant at the tropical and subtropical sites, where it comprised up to 38% of all diatoms at Darwin, 25% at North Stradbroke Island and 29% at Rottnest Island and Yongala (Kruskal-Wallis, *p* < 0.001; Figure 3, Tables S9 and S10). Among other dominant diatoms (genus found with relative abundance > 5% in at least three of the seven sites), members of the *Thalassiosira* genus were also present at all sites, ranging in relative abundance from 0.5% at Yongala to 22.4% at Port Hacking (Figure 3, Tables S9 and S10). Similarly, members of the *Skeletonema* genus accounted for up to 14.3% at Maria Island and 9.3% at Port Hacking but were present in much lower abundance (< 5%) at Kangaroo Island, Rottnest Island and North Stradbroke Island (Figure 3, Tables S9 and S10). Relative to other sites, the diatom community at Darwin exhibited a significantly higher proportion of *Navicula* ASVs (4.4%) (Kruskal-Wallis, *p* < 0.05) and on average, sites within temperate/subtropical environments hosted diatom communities with significantly higher proportions of ASVs belonging to the *Pseudo-nitzschia* genus (21.8% at North Stradbroke Island and 11% at Kangaroo Island) (Kruskal-Wallis, *p* < 0.05) (Figure 3, Tables S9 and S10).

**Figure 4:**
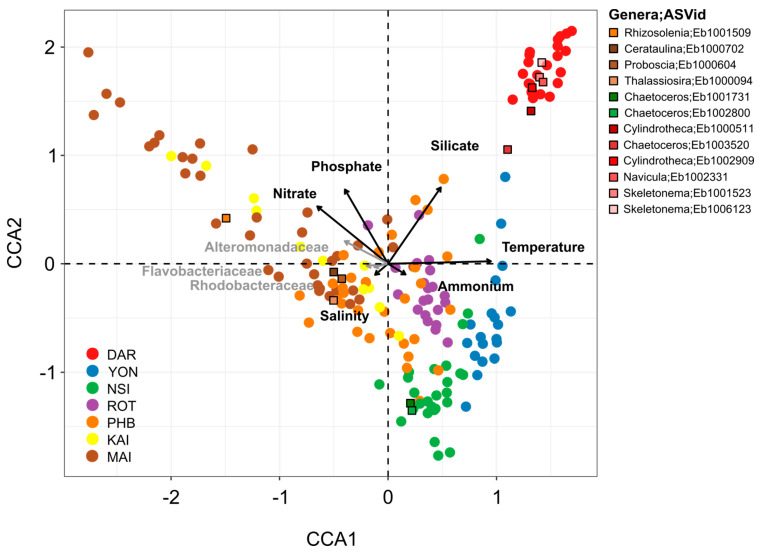
Environmental (nutrient, temperature, and salinity) and Biological (Rhodobacteraceae, Flavobacteriaceae, Alteromonadaceae) Drivers of Diatom Community Composition at Seven Sites around Australia. Diatom community composition over 5 years displayed using Canonical Correspondence Analysis (CCA) of rarefied diatom ASVs highlighting the environmental and biological drivers of assemblage composition. Black arrows represent the influence of each environmental variable (temperature, salinity, silicate, phosphate, ammonium and nitrate) and grey arrows correspond to the influence of three bacteria families most commonly found in association with phytoplankton (Rhodobacteraceae, Flavobacteriaceae, Alteromonadaceae). Abiotic parameters alone (site, temperature, daylength, silicate, phosphate, nitrate, ammonium, salinity) contributed to explain ~21.2% of the variance observed between diatom community whereas Rhodobacteraceae, Flavobacteriaceae and Alteromonadaceae bacteria families contributed to explain ~4.5%. Altogether, biotic and abiotic variables contribute to explain ~25.7% of variance observed between the diatom community. Squares represent diatom ASVs which were among the top 10% most abundant ASVs that were significantly differentially abundant between sites (ANCOM-BC analysis) and were positively correlated with specific bacterial ASVs (network co-occurrence analysis).

Across the seven different environments examined here, changes in overall diatom relative abundance were strongly correlated with temperature (Spearman’s correlation, *r* = 0.501, *p* < 0.001), followed to a lesser extent by nitrate (Spearman’s correlation, *r* = 0.340, *p* < 0.001) and phosphate (Spearman’s correlation, *r* = 0.252, *p* < 0.001), although the latter two parameters differentiated the diatom community structure at Maria Island from all other sites (Figure 4). Silicate (Spearman’s correlation, *r* = 0.099, *p* < 0.002) was also significantly correlated with changes in diatom relative abundance and differentiated the diatom community at Darwin compared to all other sites (Figure 4).

We next considered relationships between the diatom community structure and a potentially important, but often over-looked, biotic variable, the bacterioplankton community. Using the 16S rRNA gene dataset from each NRS site, we selected specific bacterial families previously identified to participate in reciprocal interactions with diatoms (e.g., Flavobacteriaceae, Rhodobacteraceae, and Alteromonadaceae) [17,27], and investigated co-occurrence patterns. These three bacterial families explained ~4% of the variation observed in diatom communities between the different sites, with temperate locations (i.e., Port Hacking, Maria Island, Kangaroo Island) characterised by a higher proportion of Alteromonadaceae, Flavobacteriaceae and Rhodobacteraceae than tropical sites (e.g., Darwin, Yongala) (Figure 4). Furthermore, at the community level, Spearman’s correlation also revealed that diatom relative abundance was strongly correlated with bacterial relative abundance (*r* = 0.447, *p* < 0.001). Cumulatively, the 10 biotic and abiotic variables considered here explained approximately 25% of the total variance observed within diatom community composition across environments (Figure 4).

A total of 221 ASVs exhibited differential abundance patterns between sites (ANCOM-BC, Table S11), including *Skeletonema ardens* (Eb1001523) and *Cylindrotheca closterium* (Eb1006609), which were principally responsible for differentiating Darwin and Yongala from all other sites (Figure 3 and Figure 4). Both *Cerataulina pelagica* (Eb1000702) and *Guinardia delicatula* (Eb1000659) were most responsible for discriminating Port Hacking and Maria Island diatom communities from the five other sites, while *Chaetoceros pseudocurvisetus* (Eb1001731) had strong influence on differentiating the North Stradbroke Island and Port Hacking diatom communities from all others (Figure 3 and Figure 4).

### 3.2. Diatom Communities Exhibit Dissimilar Levels of Seasonality According to Location

**Figure 5:**
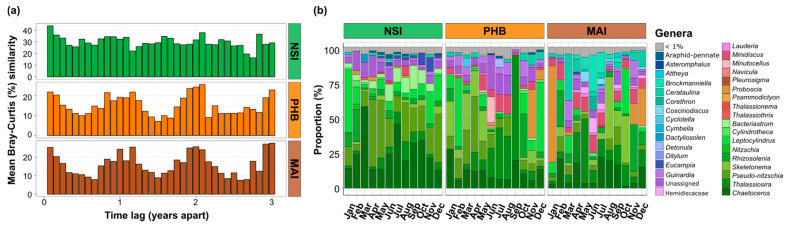
Interannual Variability of Diatom Communities (**a**) and Temporal Marine Diatom Composition (**b**). (**a**) Mean Bray-Curtis similarities of all-pairs samples separated by a given numbers of months apart. The first bar represents the mean Bray-Curtis similarity between samples that are one month apart, the second bar the mean Bray-Curtis similarities between samples that are two months apart (etc.). (**b**) Temporal community composition of the most abundant marine diatoms genera (>1% threshold) in the surface waters (2 m) at Maria Island, Port Hacking and North Stradbroke Island NRS sites across all sampling times between 2017 and 2019. Relative abundance represents the average relative abundance (surface water) of each month across the three years (2017–2019). The full dataset is presented in Figure S4.

We examined seasonal patterns in diatom community dynamics at the three NRS sites with the most complete datasets between 2017 and 2019 (i.e., Maria Island, Port Hacking and North Stradbroke Island). At Port Hacking and Maria Island, diatom community composition demonstrated a clear signal of seasonality, whereby average Bray-Curtis similarity of diatom community structure was significantly higher between samples taken one year apart (17–18%) than samples taken 6 months apart (11–12%) (Kruskal-Wallis, *p* < 0.001, Figure 5a, Table S12). At North Stradbroke Island, diatom communities exhibited a much weaker seasonal signature than Maria Island and Port Hacking, with average Bray-Curtis similarities of 27% between samples taken one year apart, compared to 23% for samples taken 6 months apart (Kruskal-Wallis, *p =* 0.034, Figure 5a, Table S12).

**Figure 6:**
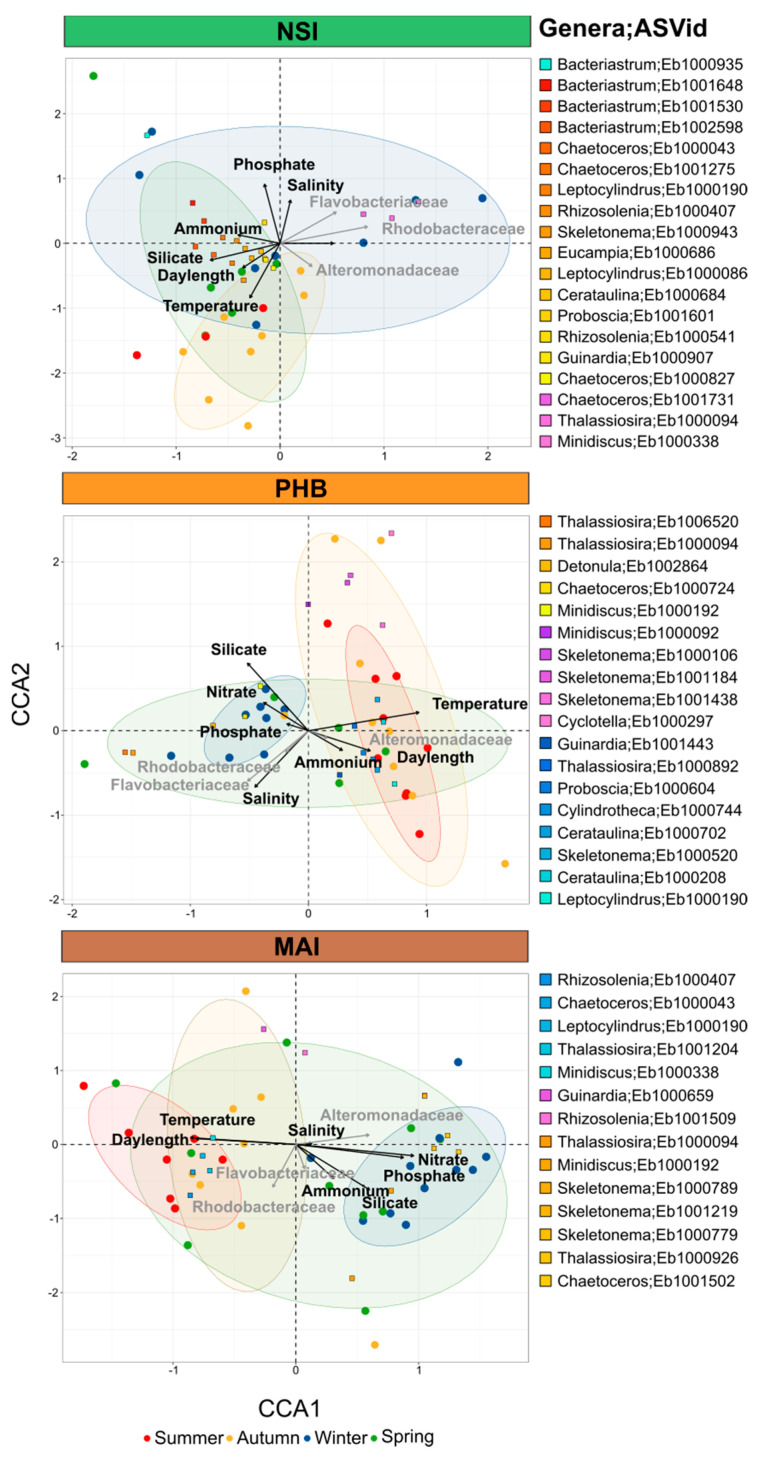
Seasonality of Diatom Communities at North Stradbroke Island, Port Hacking and Maria Island. Diatom communities over 5 years displayed using Canonical Correspondence Analysis (CCA) of rarefied diatom ASVs, highlighting the environmental and biological drivers of seasonality. Black arrows represent the influence of each environmental variables (temperature, salinity, silicate, phosphate, ammonium and nitrate) and grey arrows correspond to the influence of three bacteria families most commonly found in association with phytoplankton (Rhodobacteraceae, Flavobacteriaceae, Alteromonadaceae). Ellipses correspond to the confidence level (80%) at which to draw the ellipse for each season (Summer = red, Autumn = yellow, Winter = blue, Spring = green).

Canonical correspondence analysis further confirmed that diatom communities grouped by season, especially at Port Hacking and Maria Island, with clear summer and winter clusters identified and shown to be influenced by a combination of biotic and abiotic factors. At Port Hacking and Maria Island, discrete diatom communities observed during winter were associated with higher concentrations of nutrients (nitrate, phosphate and silicate), while overall temperature together with daylength most strongly governed the seasonality of the diatom communities (Figure 6, Table S13). At Port Hacking, the spring and summer months were characterised by a higher proportion of Rhodobacteraceae (~11.5%) and Flavobacteriaceae (~14%) while winter had the highest proportion of Alteromonadaceae of all seasons (~0.7%). Similarly, at Maria Island, winter was characterised by six times higher proportion of Alteromonadaceae while summer and spring had higher proportions of Rhodobacteraceae (~14.2%) and Flavobacteriaceae (~13.6%) (Figure 6, Table S13).

We next identified the diatom ASVs that exhibited sudden and repeated increases in relative abundance between years (greater than 4-fold between two consecutive months at each site), to identify diatom bloom dynamics at each of the three time-series sites. A total of 48 ASVs exhibiting these dynamics were identified across the three sites, with bloom events occurring between 2 and 10 times over the three-year period. Most of the ASVs exhibiting a bloom dynamic occurred at Maria Island and Port Hacking (73% and 19%), compared to North Stradbroke Island (8%). ASVs that exhibited the highest number of blooms belonged to *Chaetoceros*, *Thalassiosira*, *Leptocylindrus* and *Minidiscus* (Figure 5b and Figure S3, Table S14). Among these, one ASV belonging to *Thalassiosira* (Eb1000094) displayed bloom dynamics at all three sites, with a relative abundance reaching up to ~90% at Port Hacking in spring (September 2018), 63% at Maria Island in autumn (April 2019) and ~21% at North Stradbroke Island during winter (August 2018) (Figure 6 and Figure S3). At North Stradbroke Island, one ASV from the *Chaetoceros* genus (Eb10017231) repetitively increased (twice) in relative abundance in winter 2017 and 2018 (reaching up to 9%) (Figure 6 and Figure S3). Similarly, at both Port Hacking and Maria Island, *Proboscia* (Eb1000604), respectively, increased three and six times reaching up to 54% (December 2017) of the diatom community at Port Hacking and 89% at Maria Island (Figure 6 and Figure S3).

### 3.3. Specific Bacterial Taxa Are Strongly Correlated with Diatoms across Space and Time

**Figure 7:**
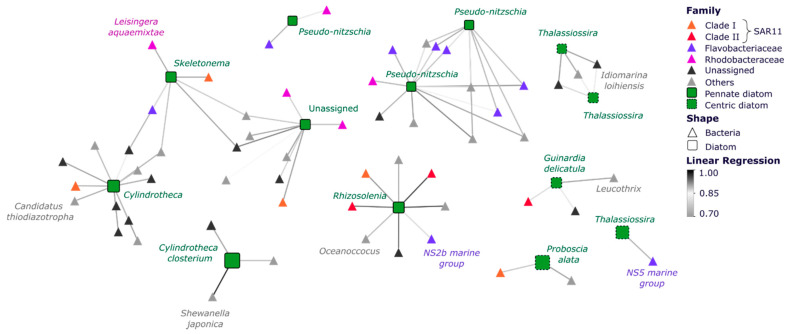
Co-occurrence Networks Subset of Diatoms and Bacteria. Correlations are based on MINE analysis and show only statistically significant positive correlations between diatom and bacterial ASVs (Linear Regression ≥ |0.7|; *p* < 0.05). Node sizes correspond to the mean relative abundance of the ASVs across the entire dataset and edge colour gradient indicates the strength of the correlation (Spearman correlation). Green squares represent diatom ASVs, whereas coloured triangles represent bacterial ASVs. The full set of significant co-occurrences is presented in Figure S5 and Tables S14 and S15.

Correlation analysis of the abundance of individual diatom ASVs with abiotic factors (temperature, ammonium, nitrate, silicate, phosphate) and concurrent bacterial ASVs identified strong co-occurrence between specific diatoms and bacteria, but notably identified no significant correlation with environmental parameters. Across the entire dataset, we identified 250 strong positive correlations between diatom and bacterial ASVs (LR > 0.7), with the majority (~65%) of these co-occurrences observed among abundant diatom genera (>1% relative abundance in at least four of the seven sites across all samples) and bacteria within Rhodobacteraceae, Flavobacteriaceae and SAR11 taxa (~13%) (Figure 7, Tables S15 and S16). An ASV belonging to the *Thalassiosira* genus and another one identified as *Cylindrotheca closterium* both exhibited co-occurrence patters with bacterial ASVs from the Alteromonadales (*Idiomarina loihiensis, Shewanella japonica*) and Flavobacteria (NS5 marine group) (Figure 7). Diatom ASVs identified as *Rhizosolenia imbricata* and *Proboscia alata* both displayed strong co-occurrence with *Oceanococcus* sp. and NS2b marine group ASVs (Figure 7). Other key diatom ASVs that displayed strong co-occurrence patterns with specific bacterial ASVs included *Skeletonema ardens* with the Roseobacter *Leisingera aquaemixtae*, *Cylindrotheca* sp. with *Candidatus thiodiazotropha* and *Guinardia delicatula* with *Leucothrix* sp.

## 4. Discussion

Diatoms are one of the most important functional groups of microbes in the marine environment [5], but also exhibit large variability in diversity and abundance over space and time [7,12]. Our study aimed to identify the main abiotic and biotic factors governing diatom community structure across a unique dataset including seven sites spanning the Australian continent. We identified temperature and nutrients (nitrate and phosphate) as key environmental determinants explaining most of the spatial variation in diatom assemblages, which was reflected in a transition of community structure from tropical to temperate climates. Diatom communities also exhibited strong seasonality at Maria Island and Port Hacking, with summer diatom communities discriminated from those occurring at other times due to higher temperature and daylength, and winter communities distinguished due to increased nutrient concentrations (phosphate, nitrate, silicate). Diatom assemblages at the temperate time-series locations at Port Hacking and Maria Island also displayed significant correlations with the putative phytoplankton-associated bacterial groups Alteromonadaceae, Flavobacteriaceae and Rhodobacteraceae. Finally, we identified key ASVs responsible for driving differences in diatom community between sites and showed that their abundance was strongly correlated with specific bacteria, further suggesting the presence of potentially important diatom-bacteria ecological interactions.

### 4.1. Environmental and Bacterial Drivers of Continental-Wide Diatom Spatial Distribution

Although each site was characterized by different diatom communities, we observed that ASVs belonging to cosmopolitan diatom genera, such as *Chaetoceros*, *Thalassiosira* or *Pseudo-nitzschia*, were regularly present across the entire dataset at all locations. This result is consistent with other large-scale studies, such as Tara Oceans which reported that *Chaetoceros* and *Thalassiosira* genera make up 23% and 13%, respectively, of all diatoms present in the global ocean [6,52]. These findings are also consistent with microscopy-based studies that have examined diatom community changes off the East Coast of Australia, where the most abundant diatom species belong to the *Chaetoceros*, *Thalassiosira* or *Pseudo-nitzschia,* and *Leptocylindrus* genera [33,53]. Our study, however, identified specific diatom ASVs that contributed to the partitioning of diatom communities between sites. For example, the centric diatom *Palmerina* is typically associated with warm tropical or sub-tropical water and high-silica conditions [54], which is consistent with its higher prevalence at Darwin compared to the other sites. Additionally, although some diatom groups displayed a cosmopolitan distribution when examined at the genus level, at the ASV level there was greater evidence for distinct patterns and relationships to environmental variables between sites. These patterns highlight the ability of specific diatom genera to adapt to a wide range of environments, while others have stricter niche boundaries.

Our results reveal that variations in water temperature and inorganic nutrients (phosphate, nitrate) had the biggest effect on diatom community structure, which is consistent with previous work that used global or regional datasets [30,32]. However, we also identified significant links between diatom community structure and the relative abundance of several key bacterial families previously implicated in important ecological interactions with diatoms (i.e., Rhodobacteraceae, Flavobacteriaceae and Alteromonadaceae). Some previous studies have also demonstrated the potential importance of diatom communities in shaping bacterial communities and that shifts in diatom and bacteria community structure can be coincident [55]. Our study demonstrates spatiotemporal correlations between diatom and bacterial communities, providing in situ support for the growing idea that significant ecological relationships exist between diatoms and specific members of the marine bacterial assemblage. Our data demonstrate not only quantitative links between changes in diatom and bacteria community structure, but also highlight the relative abundance of Rhodobacteraceae, Flavobacteriaceae and Alteromonadaceae as explanatory variables behind changes in diatom community structure. Overall, our data reveal that geographical location strongly acts to shape diatom assemblages through different temperature, nutrient regime and bacterial community.

### 4.2. Bacteria Contribute to Seasonal Variability of Diatom Communities

Diatom communities displayed strong intra- and inter-annual variability on the southeast coast of Australia, similar to what has been previously observed with higher abundance of diatom in winter/spring compared to summer/autumn [33,56]. Such seasonal variability is commonly attributed to the bloom dynamics of diatoms in response to changes in nutrient concentrations and temperature. Interestingly the strength of seasonality in the composition of diatom communities differed according to location, where the northmost site considered, North Stradbroke Island, exhibited lower levels of seasonality in diatom community composition than the more temperate locations (Maria Island and Port Hacking). This is likely explained by differences in seasonal extremes in water temperature, day length, light levels and nutrients.

In addition to abiotic factors, our data also suggest that the key bacterial communities (Alteromonadaceae, Flavobacteriaceae and Rhodobacteraceae) that influenced the spatial distribution of diatom communities also influence their seasonal dynamics. Previous studies have already demonstrated that during diatom blooms, a clear succession occurs among bacterial taxa, with Rhodobacteraceae often dominating during the beginning of diatom blooms while Flavobacteriaceae become abundant during bloom collapse [50,57]. This is believed to be due to a concomitant succession of diatom-derived organic compounds, with Rhodobacteraceae degrading low molecular weight molecules while Flavobacteria metabolise larger organic compound (polysaccharides) and take advantage of dying diatoms as the bloom terminates [34,35,58]. Our results show that members of the Rhodobacteraceae, Flavobacteriaceae and Alteromonadaceae display significant co-occurrence patterns with abundant members of the diatom community, especially during spring months when diatom blooms increase in frequency. This implies the existence of diatom-bacteria ecological links, which could be either causative (e.g., mutualistic relationships) or coincidental (e.g., sharing the same environmental niche space) in nature, but we argue, given the emerging evidence from laboratory studies for the important reciprocal exchanges between diatoms and members of these bacteria groups [28], that the former is perhaps most likely.

### 4.3. Biological Factors Influence Diatom Community Structure

Co-occurrence networks revealed that key groups of diatoms were regularly strongly correlated with specific bacteria, more specifically, diatom ASVs that were largely responsible for discriminating diatom communities between locations, such as *Thalassiosira*, *Pseudo-nitzschia*, *Skeletonema* and *Rhizosolenia*, and often displayed significant correlations to the relative abundance of bacterial ASVs belonging to groups known to establish ecological relationships with phytoplankton, including members of the Flavobacteriaceae and Rhodobacteraceae. Notably, many of these diatom ASVs also displayed increases in relative abundance indicative of bloom dynamics, indicating that diatom-bacterial interactions are likely to be most pronounced under diatom bloom conditions [17,58].

Key correlations identified in our dataset included a potential relationship between NS5 marine group-*Thalassiosira* and *Polaribacter-Pseudo-nitzschia,* which is notable given the previously established diatom bloom-associated nature of those bacteria [17,34,57,59]. In each of these examples, the bacterial partner is a member of the Bacteroidetes phylum, which is widely distributed in the marine environment and can degrade complex polysaccharides, which represent a significant component of organic matter released by diatom. Their ability to degrade complex organic matter molecules and the fact that these correlations have repeatedly been observed in several time-series further suggest that those specific taxa may have evolve to specialise and thrive in a diatom-dominated bloom event environment [58,60,61]. Other important correlations identified within our dataset involved the siderophore-producing bacteria *Idiomarina* sp. and members of the *Thalassiosira* genus. Interestingly, *Idiomarina* sp. have elsewhere been shown to enhance the growth of green algae, and the correlations observed here suggest a potentially beneficial impact of this bacterium on diatoms [62]. We also found that different diatom ASVs assigned to the same genus (i.e., *Cylindrotheca* or *Pseudo-nitzschia*) exhibit co-occurrence patterns with different bacteria, potentially indicating the species-specificity of some of these interactions.

## 5. Conclusions

This is one of the first molecular-based studies to characterise the dynamics of diatom communities across Australian coastal waters and to assess the potential influence of specific bacterial families on the spatial and temporal distribution of diatoms. Our analyses revealed that diatom communities are not only shaped by environmental parameters (e.g., temperature, nutrients), as previously demonstrated, but are also likely to be strongly influenced by specific bacterial populations. Our co-occurrence analysis both uncovered potential new diatom-bacteria interactions and provides field-based confirmation for ecological relationships previously studied in laboratory settings (e.g., *Pseudo-nitzschia*-*Roseobacter*). This study both highlights the highly dynamic nature of marine microbial communities and the likely existence of important inter-species relationships in defining the seasonal and biogeographical patterns of functionally significant groups of microbes in the ocean.

## Data Availability

All genomic and meta- data analysed in this study are publicly available can be found at the Bioplatforms Australia Data Portal (https://data.bioplatforms.com/organization/about/australian-microbiome (accessed on 4 August 2020)). Scripts, key data files and workflows to reproduce figures and statistical analyses are available from GitHub (https://github.com/NineFR09/BPA_DiatomsTimeSerie.git (accessed on 16 January 2022)).

## Supplementary Materials

The following supporting information can be downloaded at: https://www.mdpi.com/article/10.3390/microorganisms10020338/s1, Figure S1: Marine diatom composition of all depth (0–100 m) collected from seven National Reference Stations around Australia; Figure S2: Marine Diatom alpha diversity indices for the seven National Reference Stations; Figure S3: Marine diatom ASVs exhibiting repeated increase in relative abundance; Figure S4: Temporal marine diatoms community composition of the surface waters (2 m) at Maria Island, Port Hacking and North Stradbroke Island National Reference Stations; Figure S5: Full co-occurrence networks of diatom and bacterial ASVs; Table S1: National Reference Station location details; Table S2: National Reference Station environmental variables details; Table S3: Diatom surface waters 18S rRNA metadata, Table S4: Bacteria surface waters 16S rRNA metadata (0 m only and year 2015–2019); Table S5: Wilcoxon rank sum statistic test results of the comparison of environmental parameters between all sites; Table S6: Wilcoxon rank sum statistic test results of the comparison of diatom richness and diversity between all sites; Table S7: Diatom surface waters alpha diversity indices using rarefied data (to 20,000 reads); Table S8: PERMANOVA pairwise comparison of diatom community between sites; Table S9: Figure 1 diatom relative abundance data; Table S10: Figure 1 colour palette associated diatom genus data; Table S11: ANCOM-BC analysis of diatoms surface waters ASV between 2015–2019; Table S12: Kruskal-Wallis statistic test results of the difference between six month and yearly sampling time Bray-Curtis dissimilarity (%); Table S13: Kruskal-Wallis statistic test results of the comparison of environmental parameters between season at Port Hacking, Maria Island and North Stradbroke Island; Table S14: Diatom ASVs exhibiting a 4-fold increase in relative abundance at North Stradbroke Island, Port Hacking and Maria Island; Table S15: Network nodes table; Table S16: Network edges table.
