# Supplementary material for: Diatom Biogeography, Temporal Dynamics, and Links to Bacterioplankton across Seven Oceanographic Time-Series Sites Spanning the Australian Continent
Source: Microorganisms. 2022 Feb 1;10(2):338. doi: 10.3390/microorganisms10020338 (PMC8880096; doi:10.3390/microorganisms10020338)
Supplement: Supplementary file 1 [file microorganisms-10-00338-s001.zip › LeReun_etal_SupplmentaryFigures&Tables.pdf]

Diatom biogeography, temporal dynamics, and links to bacterioplankton across seven oceanographic time-series sites spanning the Australian continent

Nine Le Reun[1], Anna Bramucci[1], James O'Brien[1], Martin Ostrowski[1], Mark V. Brown[2], Jodie Van de Kamp[3], Levente Bodrossy[3], Jean-Baptiste Raina[1], Penelope Ajani[4], and Justin Seymour[1*]

[1] Climate Change Cluster, University of Technology Sydney, Ultimo, NSW 2007, Australia; nine.m.lereun@student.uts.edu.au (N.L.R.); anna.bramucci@uts.edu.au (A.B.); james.obrien@student.uts.edu.au (J.O.); martin.ostrowski@uts.edu.au (M.O.); jean-baptiste.raina@uts.edu.au (J.-B.R.)

[2] School of Environmental and Life Sciences, The University of Newcastle, Callaghan, NSW 2308, Australia; oceanmicrobes@gmail.com

[3] Oceans and Atmosphere, Commonwealth Scientific and Industrial Research Organisation, Battery Point, TAS 7004, Australia; jodie.vandekamp@csiro.au (J.V.d.K.); lev.bodrossy@csiro.au (L.B.)

[4] School of Life Sciences, University of Technology Sydney, Ultimo, NSW 2007, Australia; penelope.ajani@uts.edu.au

*Correspondence: justin.seymour@uts.edu.au

Supplementary figures

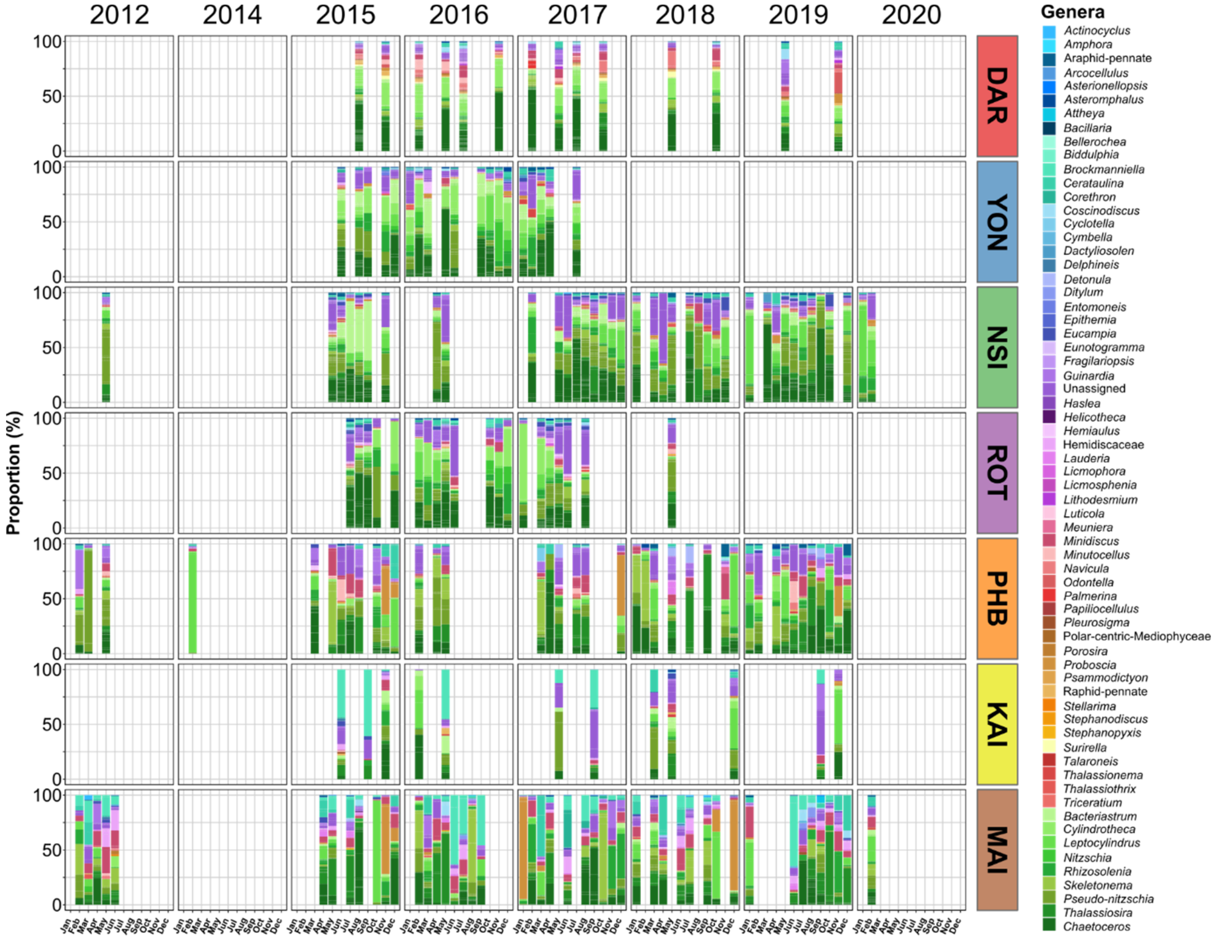

Figure S1: **Marine diatom composition of all depth (0-100 m) collected from seven National Reference Stations around Australia**. Relative abundance (%) of diatom genera across all sampling points between 2012-2020, using 18S rRNA gene as a phylogenetic marker. Non-italic text corresponds to higher taxonomy levels.

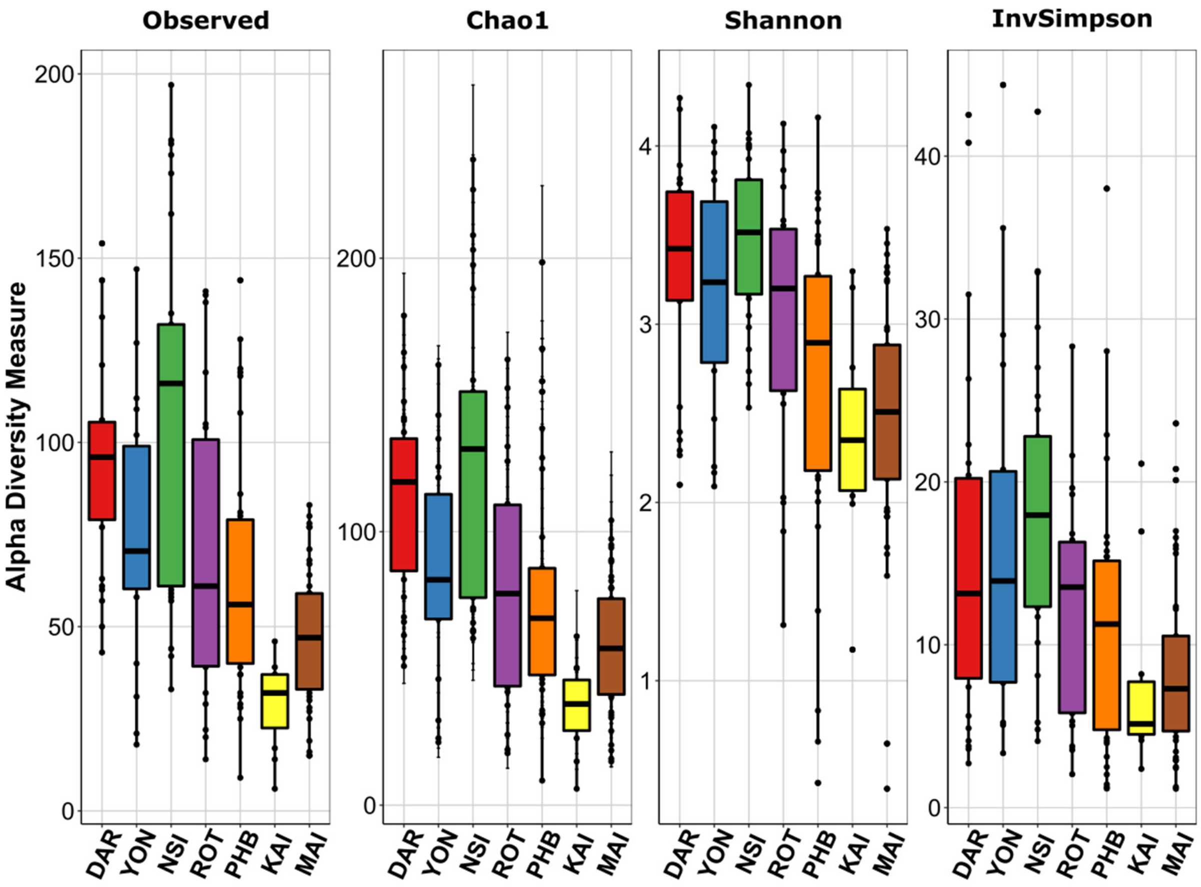

Figure S2: **Marine Diatom alpha diversity indices for the seven National Reference Stations**. Include the Observed richness, Shannon, Chao1 and Simpson indices of the surface water diatom community using rarefied data to 20 000 reads.

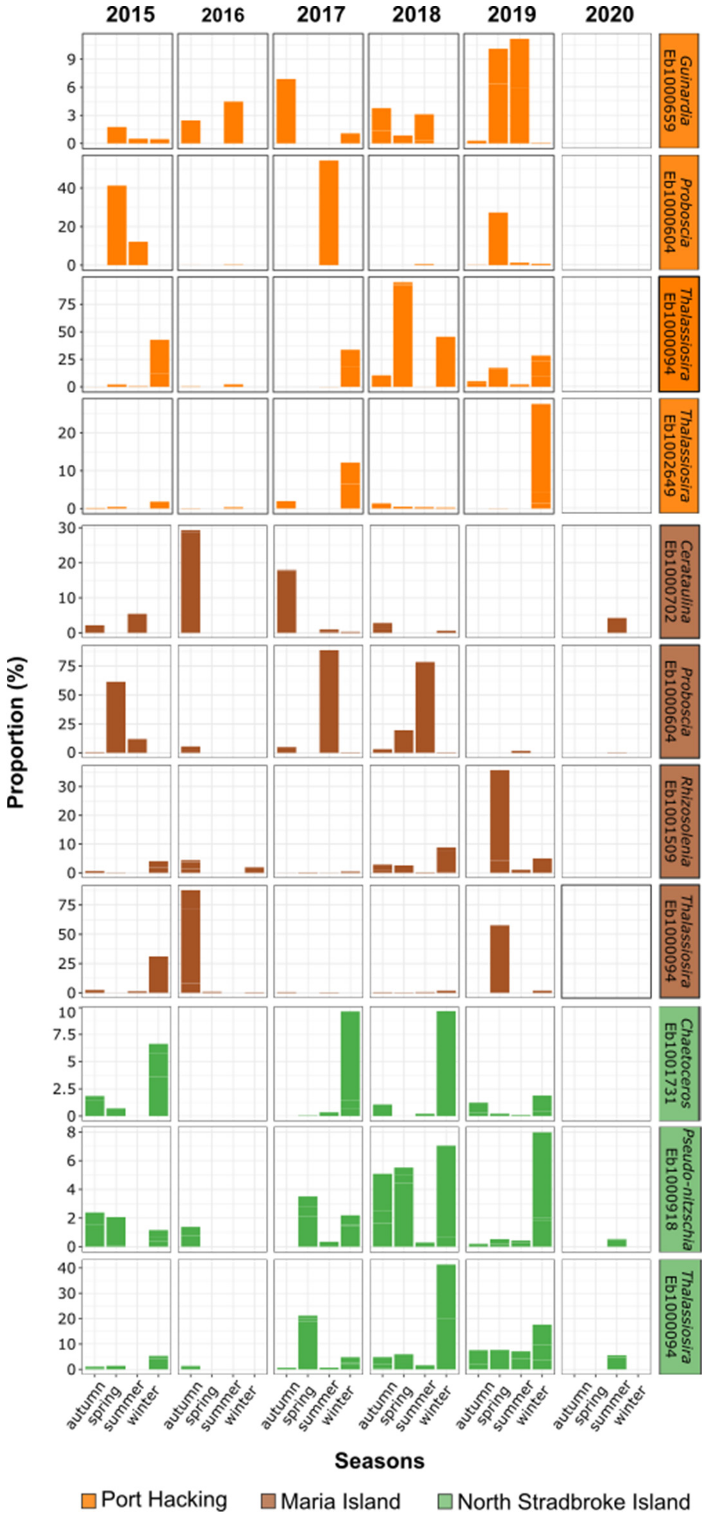

Figure S3: **Marine diatom ASVs exhibiting repeated increase in relative abundance.** Relative abundance (%) of ASVs by season across all sampling point between 2015-2020. Summer corresponds to December-January-February months, Autumn to March-April-May, Winter to June-July-August and Spring to September-October-November.

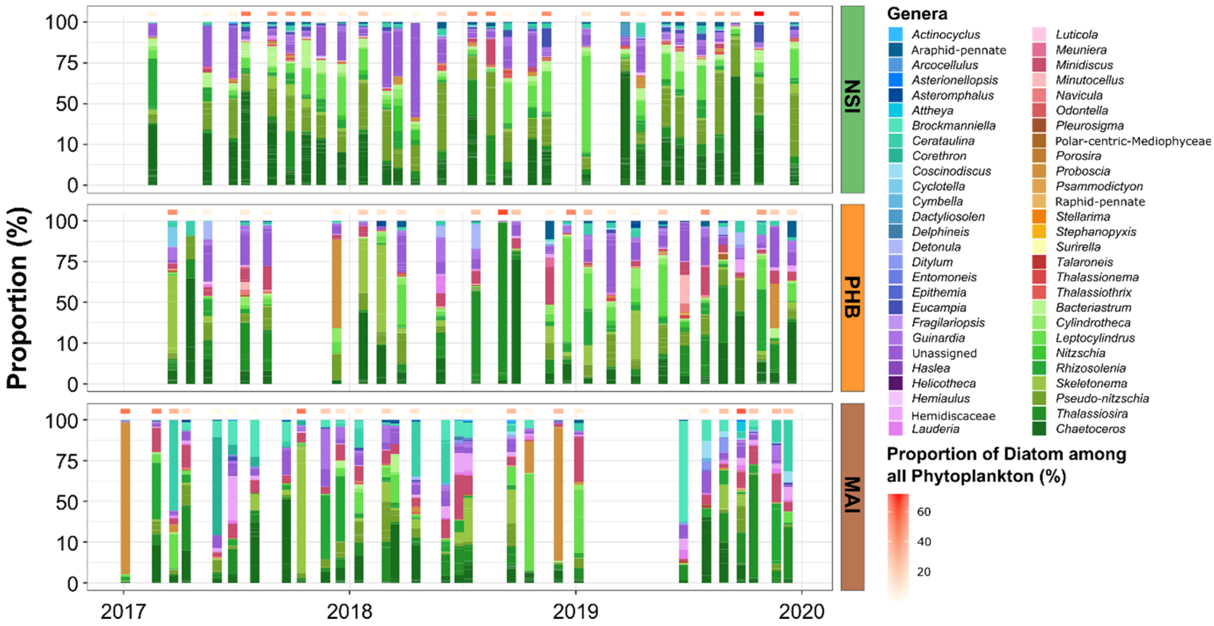

Figure S4: **Temporal marine diatoms community composition of the surface waters (2m) at Maria Island, Port Hacking and North Stradbroke Island National Reference Stations.** Relative Abundance (%) across all sampling points between 2017 and 2019. Only assigned genera with a relative abundance >1% are shown. Empty spaces denote the absence of samples and non-italic text corresponds to higher taxonomy levels. Heatmap correspond to the proportion of diatoms among all 18S phototrophs.

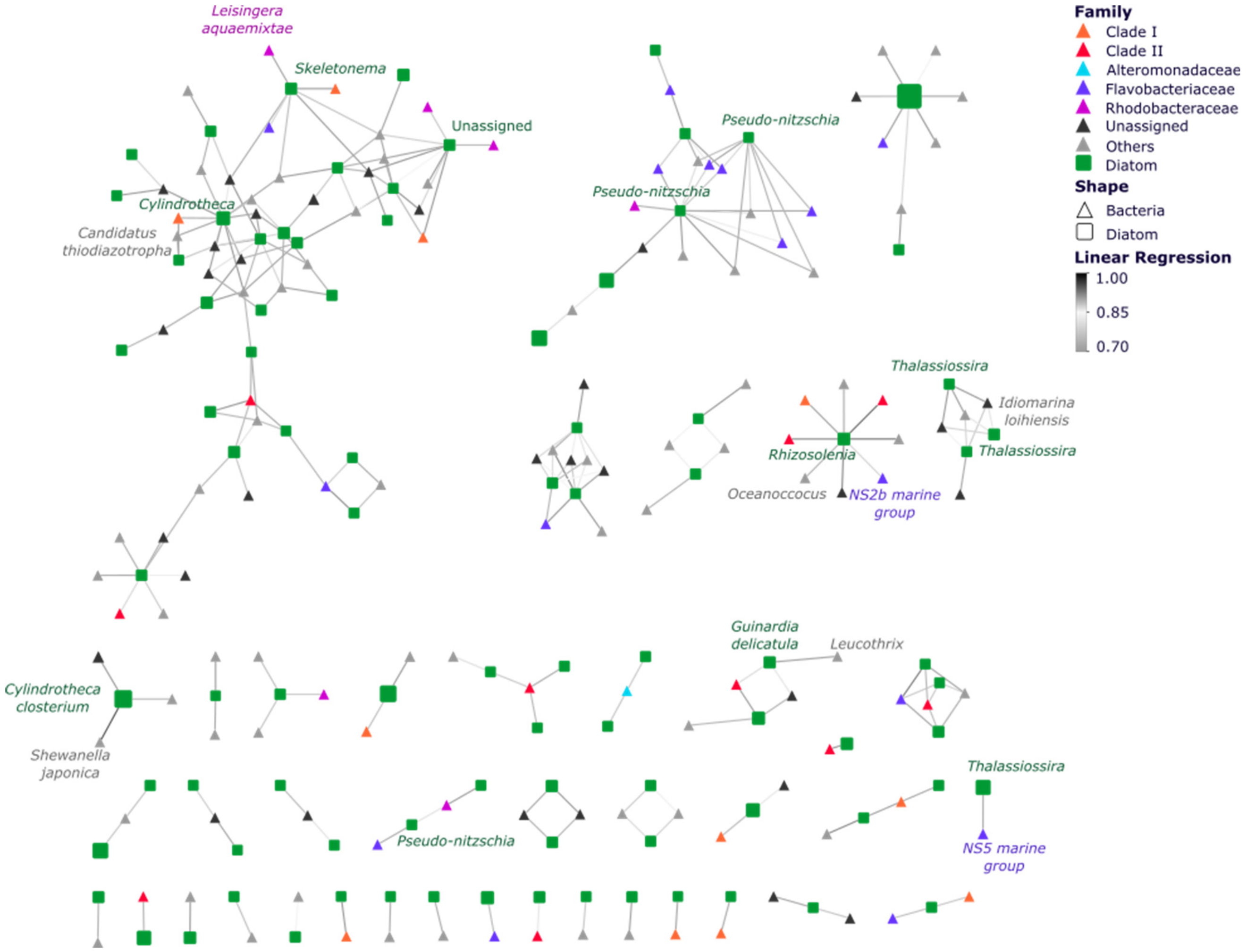

Figure S5: Full co-occurrence networks of diatom and bacterial ASVs. Correlations are based on MINE analysis and show only statistically significant positive correlations between diatoms-bacteria ASVs (Linear Regression ≥ |0.7|; $p < .05$). Node sizes correspond to the mean relative abundance of the ASVs across the entire dataset and edges colour gradient to the strength of the correlation (Spearman correlation). Green squares represent diatom ASVs, whereas coloured triangles represent bacterial ASVs. Clade I & Clade II belong to SAR11 order.

Supplementary tables

Table S1: National Reference Station location details.

Table S2: National Reference Station environmental variables details.

Table S3: Diatom surface waters 18S rRNA metadata.

Table S4: Bacteria surface waters 16S rRNA metadata (0m only and year 2015-2019).

Table S5: Wilcoxon rank sum statistic test results of the comparison of environmental parameters between all sites.

Table S6: Wilcoxon rank sum statistic test results of the comparison of diatom richness and diversity between all sites.

Table S7: Diatom surface waters alpha diversity indices using rarefied data (to 20 000 reads).

Table S8: PERMANOVA pairwise comparison of diatom community between sites.

Table S9: Figure 1 diatom relative abundance data.

Table S10: Figure 1 colour palette associated diatom genus data.

Table S11: ANCOM-BC analysis of diatoms surface waters ASV between 2015-2019.

Table S12: Kruskal-Wallis statistic test results of the difference between six month and yearly sampling time Bray-Curtis dissimilarity (%).

Table S13: Wilcoxon rank sum statistic test results of the comparison of environmental parameters and Alteromonadaceae, Flavobacteriaceae and Rhodobacteraceae between season at Port Hacking, Maria Island and North Stradbroke Island.

Table S14: Diatom ASVs exhibiting a 4-fold increase in relative abundance at North Stradbroke Island, Port Hacking and Maria Island.

Table S15: Network nodes table.

Table S16: Network edges table.
